# Why Were Zebras Not Domesticated? A Review of Domesticability Traits and Tests of Their Role in Ungulate Domestications with Macroevolutionary Models

**DOI:** 10.3390/ani14162355

**Published:** 2024-08-14

**Authors:** Netzin G. Steklis, Mateo Peñaherrera-Aguirre, Horst Dieter Steklis, Isabel Herrera

**Affiliations:** Human-Animal Interaction Research Initiative, School of Animal and Comparative Biomedical Sciences, University of Arizona, Tucson, AZ 85721, USA; nsteklis@arizona.edu (N.G.S.); irherrera@arizona.edu (I.H.)

**Keywords:** human-animal interactions and relations, domestication pathways, domesticability, ungulate domestication, carnivore-equid coevolution, ungulate capture myopathy

## Abstract

**Simple Summary:**

Beginning with Darwin, scientists have long considered the reasons for why some species but not others were domesticated. For example, of the equid species, horses and asses, but not the zebra, were domesticated. Many reasons for the ‘domesticability’ of mammalian species have been proposed (e.g., diet, social organization, response to humans). However, none of these traits have been subjected to empirical investigation. Here, we review past proposals for several domesticability traits and use a novel quantitative and evolutionary analysis to evaluate the explanatory power of these traits for domestication. For hoofed mammals, a heightened physiological response to humans, modeled as vulnerability to capture myopathy, emerged as the only significant obstacle to domestication. This is consistent with the reconstructed evolution of capture myopathy in ungulates, especially equids, and the prehistory of predation on equids, which shows that ancestral horses lost the vulnerability to capture myopathy consequent to a reduction in predation pressure on horses. Zebras, however, retained the vulnerability to capture myopathy as an adaptation to persistent high predation pressure, which would have impeded their domestication. Our results point to the importance of considering an animal’s behavior and biology as either an impediment or facilitator of domestication or developing a human-animal symbiotic relationship.

**Abstract:**

Since Darwin, many evolutionary and behavioral researchers have considered the role of phenotypic traits that favor the domestication of nonhuman animals. Among such proposed traits are a species’ social structure, level of intra- and interspecific agonistic interactions, sociosexual behaviors, parental strategies, reaction to humans, habitat preference, dietary habits, developmental trajectories, and utility to humans. However, little to no comparative phylogenetic evidence exists concerning the importance of these attributes for the domestication of animals. Moreover, rather than considering domestication as a dichotomous event (non-domesticated vs. domesticated), humans and their potential domesticates encountered numerous socioecological challenges/obstacles during the domestication process before reaching the stage of full domestication. The present study explored the influence of adult body mass, gregariousness, dietary breadth, and reaction to humans on the domestication process of ungulates. The phylogenetic comparative model revealed that capture myopathy (CM), as a proxy for reaction to humans, negatively and significantly influenced the domestication process. The present paper also explored the evolution of CM in equine species in response to the presence of large carnivoran predators during the Pleistocene. Ecologies that preserved most of the large carnivoran predators of equine species also featured more equine taxa with CM (e.g., zebras), which were thus less suitable for domestication.

## 1. Introduction

The question of why some animals were domesticated by humans while other phylogenetically close wild relatives were not has been a central one in the domestication literature [1]. For example, among equids, the horse and donkey, but not the zebra, were domesticated. Indeed, we began our investigation with the question of why zebras were not domesticated despite apparent attempts in Africa by the Dutch and Germans [2]. A preliminary examination of possible socio-behavioral differences between zebras and other equids (e.g., social organization, mating system) did not point to an obvious behavioral ecological difference that could explain why zebras were not domesticable. This led us to expand our investigation to more systematically and, when possible, quantitatively test which factors best account for the domestication of a subset of mammalian species. Beginning with Darwin, the question is usually addressed by postulating that certain traits are favorable or unfavorable for a species becoming domesticated. For example, Zeder’s [3] list of favorable and unfavorable traits includes aspects of social structure (e.g., gregarious social groups vs. family groupings) and sexual behavior (e.g., promiscuous vs. monogamous mating system). While many of these proposed traits seem reasonable, it became clear that there was little empirical support for how well they predict a species’ domestication.

The main purpose of the present paper, therefore, is to test quantitatively the association of commonly postulated traits with domestication. We restricted our macroevolutionary analysis to ungulates for several reasons: Most domesticates are ungulates, relevant data are available for this clade, and there is a sufficient number of species for comparison. To properly frame our analysis, we begin with a review of previously proposed domestication traits and proceed to examine several traits as predictors of domestication.

### 1.1. Early Hypotheses of Domestication

After Darwin’s publication of *The Origin of Species* [4], he explored the generalizability of the principle of descent with modification across various taxa, including domesticated species. Darwin was among the first to consider the role of specific attributes facilitating the domestication of nonhuman animals [5]. He proposed that the complete “*subjugation*” of an organism depended on its social nature, with domesticated animals generally accepting humans as the family, group, or herd leader. Darwin also considered the role of animal fertility in that domesticable animals must be capable of reproducing under artificial conditions. Lastly, Darwin emphasized that the aforementioned characteristics were secondary to an animal’s general utility to humans, a position that later authors called into question [6].

Following Darwin, Francis Galton explored additional contributing factors facilitating animal domestication. Galton [7] noticed significant variation between species in their attachments and aversions to heterospecifics. He observed that while some taxa were comfortable congregating with other species (bison and elk, zebras, ostriches, storks, and swallows), this pattern did not generalize across clades, even under conditions where animals occupied similar ecological niches, such as deer and sheep avoiding each other. Galton argued that heterospecific aggregations relied on the animal’s reciprocal behavioral agreeableness. For example, an animal with a calm disposition would be disturbed by the proximity and companionship of an emotionally reactive one. Galton further proposed that heterospecific aggregations would more likely emerge if each species accurately interpreted the other’s behavior. These characteristics enable certain species to not only remain tolerant of other species, such as humans, but also to develop strong attachments despite neglectful and harsh treatment. Indeed, Galton [7] suggested that if, in the course of domestication, an animal did not develop an attachment to humans, then it would likely die, escape, or become “wild”. A species’ “hardiness”—its ability to survive, even under neglectful conditions—he argued, is manifested in its capacity to invade and become established in novel locations, as is the case for pigs, goats, sheep, cattle, and horses [7].

In addition, Galton recognized other key behavioral and reproductive features. For example, the degree to which few herdsmen could manage a large group of animals (i.e., “easy to tend”) increased the probability of a species becoming and remaining domesticated. For Galton, gregariousness was a crucial component of domestication, especially if this attribute coevolved with the animals’ tolerance towards humans (Galton acknowledged that cat domestication was an exception to this principle). In addition, the capacity to reproduce in captivity was a particularly critical determinant of a species’ domesticability [7], such that species, even with the necessary temperament and attachment to humans, could not be domesticated if they could not be bred in captivity. Lastly, Galton’s domestication requirements included the extent to which the animal is useful to humans. Galton proposed that the number of resources allocated to the domestication and subsequent management of animals must have some return on investment. If humans are unable to obtain a benefit and suffer a loss from such associations, domestication will not proceed [7].

### 1.2. Preadaptations for Domestication

Although not explicitly stated, Darwin and Galton implied that features of domesticability were inherent in some animals. This idea was later developed by Hale [6], who explicitly introduced the concept of behavioral preadaptations for animal domestication in his review. (The use of this older term, “preadaptation”, has been replaced with the current evolutionary construct of “exaptation” [8]) His list of behavioral preadaptations included many of the features mentioned by Darwin and Galton (e.g., gregariousness). Hale focused on the preadaptations of mammals and birds, while Darwin and Galton did not specify the taxonomic groups in their discussion of domesticability features.

Building on Hale’s work, Price [9] argued that the degree to which a species is amenable to domestication depended on its developmental plasticity in conjunction with the captive environment’s adequacy for allowing the development and crystallization of behavioral patterns compatible with animal management and husbandry practices. According to Price, a species’ “pre-adaptations for domestication” vary as a function of an individual’s ability to adapt across multiple environmental conditions (including captive settings), as evidenced by its flexible developmental trajectories. Consequently, a species’ domesticability depends on the degree of similarity between wild and captive environments. Price [9] concluded that species with many preadaptations for captivity are more likely to be suitable candidates for domestication.

Diamond [1] observed that historically, many people attempted to tame wild animals, including the implausible grizzly bear and hyena. He appears to be the first to provide a detailed list of phenotypic “obstacles” to domestication without reference to earlier work. Inspired by Tolstoy’s novel Anna Karenina, Diamond proposed the *Anna Karenina principle*, which requires that domestication is successful only if *all* deficiencies or obstacles are avoided. According to Diamond, this principle explains why multiple species of large mammals were not domesticated (e.g., peccaries, bison, zebras). Diamond identified six main obstacles to domestication. The first obstacle, *Diet*, consists of the conversion of food biomass into the consumer organism’s biomass, which entails an energetic transformation that, on average, is 10% efficient; hence, a farmer requires 10,000 pounds of fodder for a 1000-pound cow. Thus, this bioenergetic relationship imposes a considerable restriction on the domestication of large carnivores because a 1000-pound carnivore would require 10,000 pounds of livestock meat, which in turn will depend on growing 100,000 pounds of fodder. As a result, only a few moderately sized carnivoran species were successfully domesticated (e.g., dog, cat, ferret, fox). Additionally, dietary preferences, even among herbivorous or omnivorous species, also impose constraints regarding the likelihood of successfully feeding these taxa in captivity. The second obstacle, *Growth rate*, does not favor investing in an organism with a slow growth rate, while fast-growing species would have been preferred as potential domesticates (for more recent contrary data, see [10]). The third obstacle, *the Problem of captive breeding*, refers to difficulties with a wild species successfully mating and siring offspring in a captive setting. The fourth obstacle, *Nasty disposition*, considers the species’ average aggressive, temperamental disposition. For example, Diamond [11] claimed the zebra’s “incurably vicious disposition” was an obstacle to its domestication. According to Diamond, in general, because large wild mammals are dangerous to humans, they are less domesticable, although he acknowledged that several domesticated species have been known to occasionally attack or kill humans (pigs, cattle, horses, and camels). Related to the previous point, the fifth obstacle, the *Tendency to panic*, entails a species’ average fearful disposition, often fleeing when facing a threat. Such reactions limit the probability of capturing and keeping these taxa in captivity. Lastly, a species’ *Social structure* also mediates the likelihood of domestication. Species unsuitable for domestication are often solitary, do not maintain a well-established hierarchical social organization, and the species’ herds inhabit mutually exclusive territories instead of overlapping home ranges.

More recently, Zeder [3] does not refer to Darwin, Galton, nor to Diamond’s obstacles to domestication but builds on the work of Hale [6] and Price [9,12] in proposing a suite of adaptive attributes (or “favorable characteristics”) for animal domestication. Zeder identified five main behavioral clusters, although their functional relevance to domestication is not made explicit: (1) social structure, (2) sexual behavior, (3) parent-young interactions, (4) response to humans, and (5) feeding behavior and habitat choice. Although she does not specify to which taxa these features apply, they appear to be most applicable to mammals and birds. Regarding social structure, the author argued that domesticable species exhibit large gregarious social groups with a hierarchical structure, and males are included as members of the social group. Such species also have a promiscuous mating system, whereby males are dominant over females, and movement and posture are used as sexual signals. Concerning parent-young interactions, social bonds are established through imprinting, with females accepting their offspring immediately after hatching or parturition and offspring developing precocially. Domesticable species also display a short flight initiation distance in response to human proximity, have a low behavioral and emotional reactivity to environmental changes and human interactions, habituate easily, and attract human attention by maintaining proximity. Lastly, Zeder argues that animals amenable to domestication are omnivores or dietary generalists, tolerant to multiple environmental conditions, and do not seek shelter.

### 1.3. Capture Myopathy and Its Potential Role in Hindering Domestication

Zeder [3] and Diamond [1] emphasized that one of the key factors associated with animal domestication corresponds to the animals’ physiological, behavioral, and emotional responses to human capture and management. Ungulates, in particular, are susceptible to a condition known as capture myopathy (CM) [13] that may well have interfered with attempted domestication. As described by Blumstein and colleagues, CM consists of an array of physiological sequelae (paralysis, ataxia, metabolic alterations, and rhabdomyolysis) associated with severe damage of cardiac muscle due to an animal being chased, captured, or manipulated. Moreover, emotional phenomena (such as panic, fear, and distress), above and beyond experiencing pursuit or handling, operate as risk factors, increasing the likelihood of experiencing CM. Histological signs of CM include cardiomuscular regions with necrosis and the presence of capillary microthrombii in muscular and skeletal areas [13]. Blumstein and collaborators’ review of the literature [13] indicated that CM is often classified under an array of physiological disorders, including muscular dystrophy, white muscle disease, idiopathic muscular necrosis, stress myopathy, overstraining disease, cramp, and capture paresis, among others.

Blumstein et al. [13] focused their examination on ungulates, both artiodactyls and perissodactyls, due to the amount of empirical literature on CM and life history traits. The authors showed that CM is associated with evolved high running speed and the corresponding acute physiological response to avoid predation. As life history traits are known to coevolve with socioecological indicators, Blumstein et al. argued that ungulate species congregate in groups in response to predation pressures, resulting in extended longevity. Further, group living imposes an array of daily challenges, such that larger and more complex ungulate societies are predicted to promote the evolution of larger brain volumes associated with sophisticated cognitive abilities required to navigate a dynamic social landscape.

Combining all factors, several phylogenetic logistic regression models revealed that total brain mass, maximum running speed, minimum group size, and maximum longevity positively predicted CM in ungulates. In contrast, litter size, gestation period, maximum group size, body mass, age at sexual maturity (male and female values examined separately), weaning age, body length, and litter per year did not have any significant influence on the association between life history indicators and CM. Blumstein and colleagues’ study provided partial evidence concerning the role of a slow life history strategy in CM, with maximum longevity and a larger brain volume as the only significant associations with the evolution of CM. The authors also performed an ancestral character reconstruction on CM values. Their analysis indicated that the CM gaining rate (a trait evolving in a taxon) in ungulate lineages occurred twice as fast as that of the CM losing rate (a trait disappearing in a taxon). Moreover, the reconstruction revealed that CM was evolutionarily labile, emerging, and fading across lineages.

As CM has often been viewed as a physiological response to prolonged capture and handling [13], it is reasonable to consider this cardiac pathology in preventing the domestication of several ungulates in an evolutionary analysis. It is worth noting that the current ethological and physiological literature on the correlation between CM and anti-predator behavior in ungulates is relatively scarce. Consequently, this study provides a tentative evolutionary reconstruction to be tested in subsequent phylogenetic comparative models. First, ungulates living alongside an array of large predators (including ambush and cursorial strategists) and experiencing high predation risk evolved an array of anti-predator behaviors, including greater vigilance, a more aggressive temperament, an increased flight initiation distance, and faster running speed. These behaviors are expected to be correlated with CM as they include a proclivity for stress and the necessity to flee from a source of threat. Second, as the diversity of large mammalian predators decreased in Northern Eurasia and the Levant during the Pleistocene-Holocene extinction, ungulates inhabiting locations with low predation risk relied less frequently on the latter anti-predator behaviors, thereby reducing their vulnerability to CM. Third, Biogeographical regions (e.g., the Afrotropics), where large mammalian predators persisted (including several hominin species), continued to exert considerable selective pressures on the anti-predator behaviors of ungulates and thus the accompanying risk of suffering CM. This, in turn, reduced the likelihood that populations of endemic humans would successfully domesticate these animal taxa. Lastly, Northern Eurasian and Levantine environments where ungulates survived the Pleistocene-Holocene extinction and did not coevolve with hominin populations for a prolonged period, were less likely to experience intense distress, suffer from CM, and consequently were better candidates for domestication.

In sum, since Darwin, several researchers have proposed an array of traits considered to be favorable for or obstacles to domestication. Table 1 provides a summary of the phenotypic attributes discussed in previous sections. It is remarkable that, to this day, little attention has been given to an empirical evaluation of these putative obstacles to or traits favoring domestication. Hence, the present work seeks to remedy this lack of empirical support using macroevolutionary comparative models to explicitly examine the relationship between these characteristics and animal domestication.

**Table 1:** Review of favorable phenotypic characteristics for domestication.

Domain	Indicators	Darwin [4]	Galton [7]	Hale [6]	Diamond * [1]	Price [9]	Zeder [3]
**Social structure**	Dominance hierarchy			x	x	x	x
	Sizeable gregarious social groups		x	x	x	x	x
	Males part of social group			x		x	x
	Overlapping group home ranges				x		
**Intra- and interspecies** **agonistic behavior**	Low aggression		x		x	x	
**Sociosexual behavior**	Promiscuity			x		x	x
	Males exert dominance over females			x		x	x
	Males initiate sexual advances					x	
	Postures and movements associated with sexual behavior			x		x	x
**Parental strategies**	Precocial offspring			x		x	x
	Imprinting, critical periods			x			x
	Females accept unrelated young after birth			x			x
	Offspring are easy to separate from parents					x	
**Reactions to humans**	Proclivity for Habituation					x	x
	Proclivity for Tameness		x			x	
	Short flight-initiated distances			x		x	x
	Not disturbed by human activities	x		x			x
	Easy to control		x			x	
	Solicit attention					x	x
	Low tendency to panic				x		
	Low acute response to environmental cues about potential threats (e.g., lack of capture myopathy)					x	
**Locomotion and habitat**	Restricted agility			x		x	
**preference**	Limited sensitivity to environmental changes			x		x	x
	Do not seek shelter					x	x
	Small home range					x	
**Feeding**	Omnivorous or generalist			x	x	x	x
**Development**	Fast growth rate				x		
**Breeding**	Breeds in captivity	x	x		x		
**Utility**	Usefulness to humans	x	x				

**Note**. Diamond’s * six phenotypic obstacles for domestication were reversed to transform them into favorable traits.

### 1.4. Domesticability and Domestication Pathways

**Figure 1:**
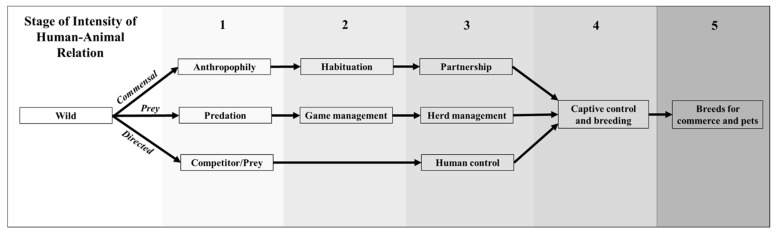
Adapted domestication pathways models based on Larson and Burger [15] and Zeder [3]. Five stages in the model correspond to the intensity of human-animal relations, with higher scores indicating higher intensity. The diagram also displays the number of transitions required to attain stage 4 on the intensity of the human-animal relation continuum per domestication pathway. The *commensal* and *prey pathways* feature three transitions before attaining stage 4. In contrast, the *directed pathway* features two transitions.

In addition to postulating domestication traits, researchers have also proposed domestication pathways (stages leading to domestication). In the following section, we review the current literature on domestication pathways with the goal of operationalizing a continuous variable for use in our subsequent analysis. Zeder [3] proposed that the process of domestication involved one of three main pathways: *commensal*, *prey*, and *directed*. As we describe below, the *commensal* and *prey pathways* involve more stages than the *directed pathway* (see Figure 1). Instead of operationalizing domestication as a dichotomous phenomenon (non-domesticated vs. domesticated species), we developed a scale to test if particular phenotypic traits explain an animal’s domesticability based on the number of stages required to reach full domestication. In other words, each stage in a domestication pathway can be seen as an ecological challenge to the symbiotic pair (i.e., humans and the prospective domesticate). Thus, the *commensal* and *prey pathways* have relatively more stages or ecological challenges with more opportunities for failure of the domestication process. Therefore, we consider species following the *commensal* and *prey pathways* to have lower domesticability potential compared to species following the *directed* pathway. We operationalize the number of stages as the Domestication Pathway Index (DPI), with the highest DPI representing ease to attain full domestication (i.e., fewer pathway stages) and the lowest DPI indicating non-domestication.

To explain the DPI more fully, we provide the following additional details about the domestication pathways. Zeder [3] proposed that in the *commensal pathway*, animals initially transitioned to interacting with humans (“anthropophily”) due to their attraction to human refuse or to consume other prey species found in human settlements. Later, some commensal animals transitioned to full “habituation” and then established a long-lasting bond with humans (“partnership”). Some examples include dogs, cats, pigs, guinea pigs, golden hamsters, chickens, Muscovy ducks, and turkeys [3]. In the *prey pathway*, ancient peoples hunted certain animal species for their hides and meat. People domesticated a subset of prey species in response to declining prey population sizes in an attempt to secure their food resources. The first transition involved “game management” or selective hunting of low reproductive value individuals (e.g., young males, old females), followed by “herd management” or husbandry of captive individuals, which eventually incorporated controlled breeding practices. Goats, sheep, cattle, zebu cattle, pigs, water buffalo, mithan, Bali cattle, yak, llamas, alpacas, and reindeer followed the *prey pathway* to domestication [3]. The third, *directed pathway*, was a more deliberate one that could be initiated only after the experience of and acquiring the knowledge from domesticating animals via the *commensal* or *prey pathways*. The target for domestication was intentionally chosen because it offered a resource of particular interest (e.g., milk, transport). Directed breeding practices followed human control. Species that were domesticated following this pathway included horses, donkeys, Dromedary camels, Bactrian camels, water buffalo, ferrets, minks, silver foxes, chinchillas, emus, and ostriches [3]. Of particular note is Zeder’s comment that species following the *directed pathway* did not likely have many, or any, of the domesticability behavioral traits, as people already familiar with the process of domestication could work around traits (e.g., with improved technology) that earlier would have been impediments to domestication, a prediction we also examined in the present study.

Larson and Fuller [14] updated and expanded Zeder’s [3] initial description of the various domestication pathways. For Larson and Fuller, the *commensal pathway* was initiated when ancient humans altered their immediate environment (e.g., producing waste), attracting various nonhuman animals. According to the authors, animals displaying greater tameness, lower aggression, and refusing to flee were more likely to benefit from these modified ecologies. Larson and Fuller [14] referred to animal populations that benefit from being entrenched in human environments as *synanthropes*. Thus, *synanthropia* is an outcome of the first stage (anthropophily) of the *commensal pathway* preceding habituation, commensalism, and even, in some circumstances, mutualism (as evidenced by human-animal reciprocal exchanges [14]).

Larson and Fuller [14] acknowledged that although the initial stages of the *prey pathway* did not require direct, intentional interventions, this pathway rested on human resource management actions. According to the authors, domesticated species following this pathway featured body sizes ranging from medium to large. Due to the nature of these interactions, prey species generally avoided people, which forced ancient humans to modify their hunting practices to increase the frequency of encounters with these taxa. For Larson and Fuller, human activities favored the emergence and persistence of behavioral traits in prey that were potentially relevant to domestication (e.g., short flight-initiated distance) as hunters’ strategies shifted because of overhunting.

In contrast, for Larson and Fuller, the *directed pathway* rested on the intentional action of humans. As the *directed pathway* occurred after the domestication of other animals via the *commensal* and *prey pathways*, ancient farmers managed and eventually domesticated wild taxa by adapting and generalizing practices developed over hundreds, and even thousands of years, to the domestication of other taxa. Larson and Fuller also recognized that despite anthropogenic selective pressures acting over multiple generations of wild taxa, some species, such as gazelles and zebras, were never domesticated. In sum, Larson and Fuller’s review of the literature, as well as their elaboration of Zeder’s domestication pathways, provides an evolutionary and ecological understanding of human activities, ranging from accidental (as in the case of the *commensal pathway* and to a lesser extent the *prey pathway*) to intentional (i.e., the *directed pathway*).

Subsequently, Larson and Burger [15] added to Zeder’s domestication pathway model, proposing that each pathway varied in the intensity of the human-animal relation continuum (IHARC). Thus, wild species displayed the lowest IHARC values. In terms of the *commensal pathway*, the transitions from wild to anthropophily, habituation, commensalism, and partnership, as well as captive animal control and intensive breeding, are accompanied by an increase in the IHARC. According to the authors, a similar process occurred with the *prey pathway*, wherein IHARC values rose in tandem with the reconstructed transitions from wild to game management, herd management, extensive breeding, captive animal control, and intensive breeding. In the *directed pathway*, the shift from wild to captive animal control and intensive breeding occurred in conjunction with increasing IHARC values. Figure 1 provides an adapted and extended version of Larson and Burger’s [15] and Zeder’s models [3].

In the present study, the DPI is based on the number of changes required to attain stage 4 in the IHARC scale shown in Figure 1, wherein humans adopted captive control and breeding as part of the domestication process. Consequently, the *commensal* and *prey pathways* required three transitions to attain stage 4, whereas the *directed pathway* required two transitions. In the DPI, higher scores indicate fewer transitions to attain captive control and breeding (i.e., higher likelihood of domestication). As wild species did not experience any transitions, they were coded as 0, *commensal* and *prey* species were coded as 1, and *directed* species were coded as 2.

### 1.5. Hypotheses

As the literature describing the various phenotypic correlates of domestication often considers the additive influence of these traits, the present study considered the simultaneous contributions of multiple predictors of domestication. Although the following hypotheses are presented separately, all parameter estimates were computed simultaneously as part of a single Phylogenetic Generalized Least Squares Model. We restricted our hypotheses to those that could be tested with available quantitative data.

**H1.**  *Adult body mass positively predicts the DPI (Life history-related hypothesis)*.

**H2.**  *Gregariousness positively predicts the DPI (Social structure hypothesis)*.

**H3.**  *Dietary breadth positively predicts the DPI (Feeding hypothesis)*.

**H4.**  *Capture Myopathy negatively predicts the DPI (Reaction to humans hypothesis)*.

## 2. Methods

### 2.1. Sample

Data on adult body mass were collected from the online data repository Pantheria [16]. Information on ungulate gregariousness and diet was collected from the Pérez-Barbería and Gordon database [17]. Data on predomestication timelines were extracted from Larson and Fuller [14]. Lastly, data on CM were taken from Blumstein and colleagues ([13]; database kindly provided by D. Blumstein). A phylogenetic tree was generated using the online platform TimeTree (http://www.timetree.org, accessed on 7 of September 2023). The phylogeny included macroevolutionary information on 65 artiodactyl and perissodactyl taxa.

### 2.2. Statistical Analyses

A convergent validity test was conducted between the predomestication times (based on the earliest signs of significant interactions between humans and the eventually domesticated species obtained from [14]) and this study’s DPI scores. Because higher DPI scores entail fewer obstacles for domestication, they imply a shorter time required to attain full domestication. Hence, a negative association is expected between DPI and time of predomestication. As macroevolutionary examinations rely on data collected from several taxa that share a common ancestor, the data’s residuals are not statistically independent [18]. Consequently, the risk of incurring Type I errors and the probability of overestimating the corresponding effect sizes increases if traditional statistical approaches are used without accounting for the underlying phylogeny and the corresponding macroevolutionary dynamics [18,19]. Moreover, statistical modeling revealed that ignoring the effects of phylogenetic proximity increases the likelihood of Type II errors [19].

The data’s statistical features require phylogenetic comparative methods that consider the evolutionary history of the clades under study. An Ancestral Character Reconstruction (i.e., a maximum likelihood estimation of the evolution of a phenotypic trait; 18) was performed on the DPI scores using the *phytools* 2.3-0 package [20]. This procedure employs maximum likelihood estimation to determine the predicted value of the corresponding ancestral nodes in the phylogeny, allowing evolutionary researchers to place a heat map onto a specific phylogeny, revealing how the trait evolved across various lineages. A Phylogenetic Generalized Linear Model (PGLS) examined the influence of Log Body Mass, Diet Breadth, Gregariousness, and Capture Myopathy on DPI scores. A PGLS model examined the influence of one or more predictors on a continuous criterion after controlling for the underlying phylogenetic effects. In contrast to other phylogenetic comparative methods, such as phylogenetic independent contrasts, PGLS computes the various parameter estimates after freely calculating the model’s phylogenetic signal (a measure of traits’ degree of evolutionary conservation; 18), wherein larger values indicate that the similarity in trait estimates among species in a phylogenetic tree is in part attributable to their shared evolutionary history; in contrast, lower values suggest the similarity in trait values is due to shared ecological dynamics leading to the emergence of phenotypic homoplasies [19]. This analysis used the pgls function found in the R package caper [21].

## 3. Results

**Figure 2:**
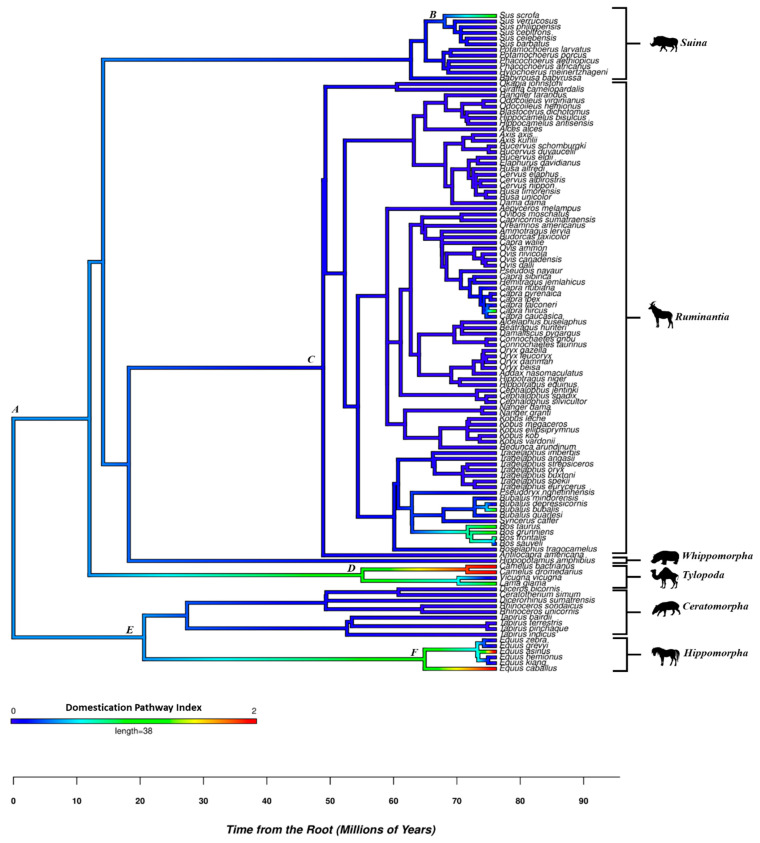
Ancestral character reconstruction of domestication pathways on a sample of various mammalian suborders with special emphasis on *Artiodactyla* and *Perissodactyla*. Silhouettes sourced from http://www.phylopic.org (accessed on 10 of October 2023). Images licensed for use under Public Domain, Creative Commons 1.0 license.

The bivariate analysis revealed a significant negative association between the domesticated species’ time since predomestication and the DPI scores (*r* = −0.58, *p* = 0.0185), offering evidence of the DPI’s convergent validity. The Ancestral Character Reconstruction provided in Figure 2 features the DPI as a continuous variable, wherein warmer colors represent higher DPI scores and colder colors have lower values in the scale. It revealed that suborders and families differed considerably in their DPI scores. Moreover, the analyses indicated that the common ancestor of artiodactyls and perissodactyls had a slight proclivity towards domestication (Figure 2, node A). This inclination facilitated the domestication of *Sus scrofa* despite the loss of this predisposition in most species within the suborder *Suina* (Figure 2, node B). Regarding the suborder *Ruminantia*, the common ancestor of taxa associated with this suborder (Figure 2, node C) features a decline in its proclivity towards domestication, with few exceptions reversing this pattern (i.e., reindeer *Rangifer tarandus*, sheep *Ovis aries*, goats *Capra hircus*, common cattle *Bos taurus*, and yak *Bos grunniens*). Moreover, the observed increase in domesticability occurred relatively recently between the Late Miocene (~10 mya) and Early Pliocene (~5 mya). In contrast to the suborder *Ruminantia*, the increased domesticability of species within the suborder *Tylopoda* (i.e., the camelids, Figure 2, node D) can be traced back to between the Mid-to-Late Eocene (~40 mya) and Early Oligocene (~34 mya). Concerning the order *Perissodactyla* (Figure 2, node E), the analysis revealed that domesticability as a trait evolved during the Late Paleocene and the Early Eocene (~56 mya), decreasing in the suborder *Ceratomorpha* and increasing in the suborder *Hippomorpha*, (Figure 2, node F). According to the analyses, the resistance to domestication observed in some hippomorph species, such as zebras, occurred relatively recently (~5 mya), suggesting that the proclivity towards domestication observed in the common ancestor of hippomorphs disappeared in some extant species of equids.

A Phylogenetic Generalized Least Squares Model (PGLS) examined the contribution of Log Body Mass, Diet Breadth, Gregariousness, and CM to DPI, revealing that the joint phylogenetic signal of the model (Pagel’s λ = 0.373) was not significantly different from zero (*p* = 0.15780) but was significantly different from one (*p* < 0.0001). This suggests that the examined traits in the statistical model did not feature a large phylogenetic signal and consequently were not conserved at the evolutionary level (i.e., the attributes evolved on multiple occasions independently of their shared evolutionary history. Hence, these traits operated as homoplasies rather than homologies). The model reached statistical significance (*p* = 0.01101) and explained 21.14% of the variance. Of the predictor variables, neither Log Body Mass, Diet Breadth, nor Gregariousness significantly predicted the DPI. In contrast, CM negatively and significantly predicted the DPI, suggesting species with a susceptibility for severe cardiac damage due to overexertion in response to capture were less domesticable. These results are further described in Table 2.

## 4. Discussion

Since the publication of the *Origins of Species* [4], evolutionary researchers have considered potential factors contributing to animal domestication. For example, Zeder [3] proposed five behavioral clusters facilitating animal domestication, including the species’ social structure, sexual behavior, parent-offspring interactions, response to humans, and feeding-habitat choice. Zeder argued that domesticable taxa were often gregarious, promiscuous, featured a dominance hierarchy, had social bonds based on imprinting, featured low behavioral and emotional reactivity to ecological shifts and anthropogenic interactions, and were either omnivores or dietary generalists. Although previous scientific publications have provided a detailed theoretical understanding of potential behavioral correlates of domestication, to our knowledge, no studies have empirically examined these associations via phylogenetic comparative modeling. Although the present study did not include all the numerous predictors of domestication that are often described in the evolutionary and ethological literature, the present analyses considered the influence of several predictors, from body size to stress (operationalized as CM) and dietary breadth.

Ancestral character reconstructions revealed noticeable differences in the domesticability across ungulate taxa. For example, within artiodactyls, the proclivity for domestication occurred as far back as the Mid-to-Late Eocene and Early Oligocene years ago for Tylopoda but considerably more recently for all other domesticated artiodactyl taxa. This result is not unexpected as humans domesticated multiple camelid species, such as Dromedary camels, Bactrian camels, and llamas, lineages that shared a common ancestor during the Late Miocene [22]. Consider two evolutionary trajectories: The first assumes that camelid domesticability evolved independently on multiple occasions (homoplasy); the second assumes that the last common ancestor (homology) of extant camelid species had attributes that increased their inclination toward domestication. The first trajectory requires multiple evolutionary steps, while the second requires fewer evolutionary changes. Hence, the second trajectory is a more parsimonious reconstruction.

The Ancestral Character Reconstruction also revealed a similar pattern within perissodactyls with *Equidae*, wherein the proclivity for the domestication of *E. asinus* and *E. caballus* (two species separated by 10 million years of evolution) originated approximately 10–20 million years ago. These results strongly suggest that the last common ancestor of these domesticated taxa scored higher on its domesticability compared to other ancient perissodactlys. Consequently, these findings reframe the persistent question concerning the non-domesticability of multiple zebra species compared to other equids. Whereas previous research [1] presupposed that the last common ancestor of contemporary equids featured low domesticability attributes akin to those featured in extant zebras, the present study indicates that the DPI of the last common ancestor of zebras, horses, and asses was higher than for contemporary zebras but lower than for contemporary horses and donkeys. In contrast to the ancient proclivity of certain perissodactyls for domestication, in most artiodactyl lineages, the proclivity for domestication was low in the common ancestor of all bovines, with an increasing DPI relatively recently.

The present study examined the influence of Body Mass, Diet Breadth, Gregariousness, and CM on the DPI. Our results from phylogenetic comparative models strongly suggest that neither Body Mass, Diet Breadth, nor Gregariousness significantly predicted the DPI. In contrast, species susceptible to CM scored lower on the DPI. This pattern indicates that ungulate species vulnerable to cardiac disorders or damage due to capture and handling are less suitable for domestication. Hence, this may help explain why horses and asses, but not zebras, were domesticated (see below for further details). To our knowledge, this is among the first phylogenetic comparative studies to consider the role of life history indicators (such as body size), dietary generalism, sociality, and physiological correlates of stress in the domesticability of ungulates. Future studies must determine whether the aforementioned predictors generalize to other animal clades, including carnivorans, lagomorphs, and rodents. Moreover, future phylogenetic comparative examinations should focus on the negative significant association between CM and domestication.

### 4.1. Predators and the Evolution of Capture Myopathy in Equids

Equids are an adaptable taxon of ungulates that have been known to change their social systems based on the environments they live in. Zebras demonstrates this well: Some zebra species have adapted to more arid environments by living in a fission-fusion social organization, while other species have adapted to areas rich in nutrients by having a harem-based social organization. Wild asses have adapted using similar living strategies, with the khulan adding to the diversity of social living by adopting a more family-based social organization to better defend against attacks from wolves. When these families gather to form a herd, multiple males are able to fend off wolves more effectively. Horses, too, change their social organization and mating system depending on the amount of forage in their area. Table 3 provides a brief summary of all the species discussed in this section and their socioecology. The available information indicates that equids (domesticated or non-domesticated) do not have substantially different social systems and, in fact, share very similar social systems with slight variations to adapt to their environments. The socioecology of the equids is such that they have a somewhat flexible social organization (grouping pattern) that switches between fission-fusion and harem groups depending on the amount and distribution of resources available and predation pressure. Likewise, their mating systems can vary between resource-defense polygyny and polygyny/single-male, as shown in Table 4.

**Table 3:** The social system of equids is described according to three dimensions: social organization, mating system, and social structure. Ecology of equids described by habitat and countries/distribution.

Equid Socioecology					
Species	Social Organization	Mating System	Social Structure	Habitat	Countries/
Plains Zebra *E. quagga* (*formerly E. burchelli*)	Harem [23,24,25,26,27,28,29,30,31,32,33,34]	Polygyny/ Single-Male [23,24,25,26,27,28,29,30,31,32,33,34]	Older members of the group initiate movement. [23,24,25,26,27,28,29,30,31,32,33,34]	Treeless grasslands and savanna woodlands [35]	Southern Sudan and southern Ethiopia, east of the Nile River, to southern Angola and northern Namibia and northern South Africa [35]
Burchell’s Zebra *E. quagga burchellii*	Same as above *	Same as above *	Same as above *	Same as above [35]	Botswana to south-east to KwaZulu-Natal and Swaziland. It is now extinct in the middle of its range [35]
Chapman’s Zebra *E. quagga chapmani*	Same as above *	Same as above *	Same as above *	Same as above [35]	North-east South Africa, north to Zimbabwe, west into Botswana, and southern Angola [35]
Grant’s Zebra or Boehm’s Zebra *E. quagga boehmi*	Same as above *	Same as above *	Same as above *	Same as above [35]	Zambia, DR Congo, Tanzania, Uganda, south-west Kenya as far as Sotik, and east Kenya, east of the Rift Valley, into southern Ethiopia and Somalia [35]
Crawshay’s Zebra *E. quagga crawshaii*	Same as above *	Same as above *	Same as above *	Same as above [35]	Occurs in Zambia, east of the Luangwa River, Malawi, south-eastern Tanzania from Lake Rukwa east to Mahungoi, and Mozambique as far south as the Gorongoza district [35]
Maneless Zebra *E. quagga borensis*	Same as above*	Same as above*	Same as above*	Same as above [35]	Ranges in north-west Kenya, from Guas ngishu and Lake Baringo, to the Karamoja district of Uganda and south-east Sudan, east of the Nile River to the northern limit of the species at 32° N [35]
Grevy’s Zebra *E. grevyi*	Fission-Fusion [23,24,25,26,27,28,36,37]	Resource Defense Polygyny/Multi-Male [23,24,25,26,27,28,36,37]	There is little to no dominance among females nor between male-female [23,24,25,26,27,28,36,37]	Arid and semi-arid grass/shrubland where there is permanent water [35]	Confined to the Horn of Africa, specifically Ethiopia and Kenya. They may persist in southern Sudan [35]
Mountain Zebra *E. zebra*	Harem [23,26,28]	Polygyny/Single-Male [23,26,28]	Dominance hierarchy exists but does not correlate with leadership [23,26,28]	Rugged mountain slopes, open grasslands, woodlands, and areas with good vegetation and perennial water sources [35]	Southern parts of South Africa through Namibia and into extreme south-western Angola (Penzhorn in press) [35]
Cape Mountain Zebra *E. zebra zebra*	Same as above *	Same as above *	Same as above *	Same as above [35]	Mountain ranges forming the southern and western edge of the of the central plateau of the Eastern Cape and Western Cape provinces of South Africa, from the Amatola Mountains in the Cathcart District westward and northward to the Kamiesberg in Namaqualand in the Northern Cape [35]
Hartmann’s Mountain Zebra *E. zebra hartmannae*	Same as above *	Same as above *	Same as above*	Same as above [35]	Mountainous transition zone between the Namib Desert and the central plateau in Namibia, with a marginal extension into south-western Angola [35]
African Wild Ass *E. africanus*	Fission-Fusion [24,26,28,38]	Resource Defense Polygyny/ Multi-Male [24,26,28,38]	Little to no dominance among females nor between male-female [24,26,28,38]	Arid and semi-arid bushland and grassland [35]	The African Wild Ass occurs in Eritrea, Ethiopia, and Somalia; some animals may persist in Djibouti, Sudan and Egypt [35]
Nubian wild ass *Equus africanus africanus* [38]	Same as above *	Same as above *	Same as above *	Same as above [35]	Lived in the Nubian desert of north-eastern Sudan, from east of the Nile River to the shores of the Red Sea, and south to the Atbara River and into northern Eritrea [35]
Donkey (D) *E. asinus*	Fission-Fusion and Harem [23,24,39,40]	Resource Defense Polygyny/ Multi-Male And Polygyny/ Single-Male [23,24,39,40]	Unstable dominance hierarchy, but there is a stable “dyad pairing” between mother and foal [23,24,39,40]	Varies between arid environments and mild environments [35]	Found globally with Ethiopia, China, and Mexico having the largest population [41]
Somali wild ass *Equus africanus somaliensis* [38]	Same as above *	Same as above *	Same as above *	Same as above [35]	Denkelia region of Eritrea, the Danakil Desert and the Awash River Valley in the Afar region of north-eastern Ethiopia, western Djibouti, and into the Ogaden region of eastern Ethiopia. In Somalia, they ranged from Meit and Erigavo in the north to the Nugaal Valley, and as far south as the Shebele River [35]
Asiatic Wild Ass *E. hemionus*	Fission-Fusion (All-female groups and territorial males) or Harem [23,24,25,37,42]	Resource Defense Polygyny/ Multi-Male or Single Male [23,24,25,37,42]	Little to no dominance among females nor between male-female Or some dominance [23,24,25,37,42]	Asiatic wild ass inhabit mountain steppe, steppe, semi-desert and desert plains. They are usually found in desert steppe [35]	Southern part of Mongolia and adjacent northern China [35]
Onager *E. hemionus onager*	Fission-Fusion (All-female groups and territorial males) [25,37]	Resource Defense Polygyny/ Multi-Male [25,37]	Little to no dominance among females nor between males-female [25,37]	Asiatic wild ass inhabit mountain steppe, steppe, semi-desert and desert plains. They are usually found in desert steppe [35]	Mongolia to Saudi Arabia and southern Russia and Kazakhstan [35]
Khur *E. hemionus khur*	Same as above [23,25,37]	Same as above [23,25,37]	Same as above [23,25,37]	Asiatic wild ass inhabit mountain steppe, steppe, semi-desert and desert plains. They are usually found in desert steppe [35]	Recent evidence of khur along the India-Pakistan border. During last two decades khur has shown range expansion along with an increase in their population [35]
Kulan *E. hemionus kulan*	Harem or all-male groups [23]	Polygyny/ Single-Male [23]	Some dominance between males and females [23]	Deltas, hot and cold deserts, semi-deserts, steppes, arid grasslands and shrublands [35]	In Mongolia, where it was formerly widely distributed throughout steppe and semi-desert habitats, from the extreme west of the country to the Mongolian-Russian-Chinese border in the extreme north-east [35]
Mongolian wild ass/khulan *E. hemionus hemionus*	Same as above [24,42]	Same as above [24,42]	Same as above [24,42]	Desert-steppe, semi-desert, desert habitats in the Gobi desert [35]	China and Mongolia [35]
Gobi kulan *E. hemionus luteus*	Same as above [23]	Same as above [23]	Same as above [23]	Same as above [35]	Southern Mongolia and northern China [35]
Kiang *Equus kiang*	Fission-Fusion [35]	Resource Defense Polygyny/ Multi-Male [35]	Unknown	Open terrain, mainly found in plains, alpine meadows desert steppes, broad valleys, and hills where grasses and sedges, are abundant [35]	Most of the distribution is in China, but the species extends into northern parts of Pakistan, India, Nepal and possibly Bhutan [35]
Western kiang *Equus kiang kiang*	Same as above [35]	Same as above [35]	Same as above *	Same as above [35]	In Pakistan, at the Oprang and Muztagh Rivers, close to the Pakistan-China border [35]
Eastern kiang *E. kiang holdereri*	Same as above [35]	Same as above [35]	Same as above *	Same as above [35]	Unknown
Southern kiang *E. kiang polyodon*	Same as above [35]	Same as above [35]	Same as above *	Same as above [35]	In India, kiang occurs in the Ladakh region of the Jammu and Kashmir state, and in northern Sikkim. In Nepal, kiang are restricted to a few areas along the border with China [35]
Horses (D) *Equus caballus*	Harem Occasionally can become a Fission- Fusion social organization [23,25,27,43,44]	Polygyny/ Single-Male [23,25,27,43,44]	The older members of the group are dominant over the younger members. [23,25,27,43,44]	Varies between arid and mild environments [44]	Found mainly in the United States and Australia [44]
Przewalski’s Horse or Asian Wild Horse *E. ferus przewalskii* [45,46]	Same as above [47,48]	Same as above [47,48]	Same as above [47,48]	Inhabit mountain steppe, steppe, semi-desert and desert plains. Usually found in desert steppe. [35]	Mongolia China, Kazakhstan and Ukraine [35]

* = There were no academic sources on this subspecies, and they are assumed to be the same as the main species. D = Domesticated.

Previous publications on domestication describe how the social system is predictive of the domesticability of a species. For example, regarding the *social organization* of equids, prominent researchers in this field [3] stated that animals with a harem social organization would have been more domesticable. While it is true that the horse and the donkey (both domesticated equids) exhibit a harem social organization, so do many undomesticated equids (Zebras, Asian Wild Asses). For example, the Plains zebra has a harem social organization, a polygyny single male mating system, and a dominance hierarchy. In these features, the Plains zebras are the same as horses. However, they are not domesticatedFurther, the horse and the donkey can adapt their grouping pattern to fission-fusion social organization, depending on the ecology, which overlaps with the social organization of other undomesticated equid species (Zebras, African Wild Asses).

Horses can switch between a harem and fission–fusion social organization, while donkeys and African wild asses both express a fission–fusion social organization (see Table 4). However, there are also some studies stating that donkeys also have the ability to switch between social organizations. Looking at their reproductive system, it would seem that the African wild asses would be more domesticable than horses, and horses would be more domesticable in terms of their social structure. However, both have been domesticated. In zebras, all three species have a mixture of both favorable and unfavorable domestication traits. Thus, the general components of a social system do not predict the domesticability of equids and indicates that domesticability is dependent on different factors, likely their susceptibility to CM.

To understand further why zebras, unlike other equids, were not domesticable because of their vulnerability to CM, we review the coevolution of equids and large carnivorans from the Pliocene to the Holocene by biogeographical regions. In this review, we were particularly interested in reconstructing the relative predator pressure on zebras versus other equids as a way of explaining the differential vulnerability to CM of zebras compared to other equids.

After sequencing the genome of several equine species (*E. ferus caballus*, E. *f. przewalskii*, *E. asinus*, and Late Pleistocene horse taxa), Orlando and collaborators [49] determined that the equine lineage leading to extant horses, zebras, and asses evolved approximately 4.5 to 4.0 mya. The authors also concluded that equine population size varied considerably in the last 2 mya as a function of climatic instability, suggesting that ecological fluctuations and predatory pressures contributed to the latter demographic declines. The analysis also suggested that extant lineages of domesticated and Przewalski horses evolved approximately 72 to 38 kya. The model revealed no hybridization between domestic and Przewalski horses, supporting the assertion that the latter lineage has remained wild throughout its evolution. More recently, Orlando [50] reviewed the paleogenomic literature and produced a phylogenetic reconstruction of horse lineages based on genomic sequencing. According to the authors, the paleogenomic data indicate that at least three main divergences occurred within the latter phylogeny. The oldest bifurcation was approximately 335 to 285 k years in Iberia. This event was followed by a second divergence of around 130 to 110 k years in Siberia. Finally, the last divergence occurred between 55 and 35 k years, with one branch leading to the rise of the Botai and Przewalski lineages and the other to the DOM2 lineage and modern breeds of horses. Orlando and collaborators hypothesized that as the Iberian lineage originated before eastern European populations, it is likely that Iberian human societies domesticated horses in Iberia before Bronze Age human communities in the Pontic-Caspian steppe. Additional paleogenomic studies are required to examine this hypothesis adequately.

Recent phylogeographic reconstructions of equids suggest that in North America, *Equus simplicidens* speciated approximately 3.5 million years ago [51]; subsequently, the genus *Equus* invaded Northern East Asian ecologies, diversifying into multiple species (now extinct), including *E. eisenmannnae*, *E. yunnanensis*, *E huanghoensis*, *E. sanmeniensis*, *E. qingyangensis*, and *E. teihardi*. Paleontologists and Paleobiologists have also described extinct *Equus* taxa in South Asia, such as *E. sivalensis* [51]. In tandem, several extinct *Equus* lineages have also been found in Italy, as in the case of *E. livenzovensis*, *E. stenonis*, and *E. stehlini*, strongly suggesting that the genus *Equus* invaded and dispersed across the Palearctic at an accelerated rate. In Eurasia, more recent lineages speciated into *E. ferus*, *E. heminous*, and *E. kiang* [52]. The oldest remains of *Equus* in East Africa are dated approximately 2.3 million years ago. Subsequently, multiple taxa speciated between 1 million and 500 k years ago, including *E. oldowayensis* and *E. koobiforensis. E. africanus*, and *E. grevyi* [51].

Concerning the distribution of and symbiotic associations between European carnivorans and equids during the Pleistocene, paleoecological reconstructions indicate that not all large mammalian predators hunted horses with the same frequency and intensity. European hyenas (*Hyaena spelaea*) arrived from Africa in Europe during the Late Pleistocene approximately 126 kyr ago, with the species disappearing from the paleontological record approximately 22 kyr ago [53]. Approximately 22% of prey bone assemblages recovered from hyena dens found near mammoth steppes corresponded to *E. caballus* (aggregate estimate of *E.c. przewalskii* and *E.c. hydruntinus*; Diedrich,53; although the authors refer to this species as *E. caballus*, the domesticated horse, the correct classification is *E. ferus*). This percentage was slightly higher close to river valleys or mountain margins, where the percentage of *E. caballus* bone assemblages ranged from 25% to 27%. In contrast, the percentage of equine remains declined in frequency in boreal regions where hyenas shared their dens with cave bears (4% to 10%; 53). Moreover, the remains of equines are nearly absent in boreal regions frequently occupied by cave bears. These results strongly suggest that whereas cave bears preferred boreal forest prey, hyenas specialized in equine hunting [53]. Concerning the diet of steppe lions, several studies based on nitrogen isotopic analyses revealed that this feline species featured a dietary specialization for *Cervus*, *Megaloceros*, *Dama*, *Bos*, and *Bison* [53]. Regional and temporal differences have also been reported with isotopic models revealing that cave lions consumed European equids during the Pleistocene, albeit not to the same extent as reindeer.

Further evidence of hyena specialization on equids comes from recent research by Dusseldorp [54]. Dusseldorp contrasted the bone assemblages associated with hyena dens and Neanderthal sites (henceforth referred to as Mousterian) in France. The author computed the average proportion of species reported in hyena dens and Mousterian bone assemblages. The statistical analyses revealed that bovids (~30% of the average proportion) and equids (~40% of the average proportion) were the predominant prey species found in hyena dens. Cervids, caprids, and megafauna occurred in fewer frequencies (less than 15% of the average proportions for each species group). In contrast, cervids were present in multiple Mousterian sites (above 55% of the average proportion). Bovids (less than 15% of the average proportion) and equids (less than 20% of the average proportion) were recovered in fewer frequencies. Dusseldorp found statistically significant differences in the average proportion of bovids, equids, cervids, and megafauna bone assemblages between hyena dens and Mousterian sites. These findings provide additional zooarchaeological evidence concerning decreased dietary overlap between hyenas and Neanderthals in France during the Pleistocene. Although the paleoanthropological and zooarchaeological records indicate Neanderthals occasionally hunted equids, hyenas were the predominant predators of these Pleistocene equids. However, this pattern changed with the arrival and spread of *Homo sapiens* across Europe, leading to the decline and extinction of multiple Pleistocene and Holocene taxa.

Recent paleomorphological studies reported that Ukrainian cave lions (*Panthera spelaea*) decreased their body size during the Late Pleistocene. Moreover, the paleontological record strongly suggests *P. spelaea* became extinct approximately 17 kyr ago. According to Marciszak and colleagues [55], the paleontological record indicates that Persian lions (*P. leo persica*) invaded the Pontic-Caspian steppe ~11.5 kyr after the extinction of *P. spelaea*. Marciszak and collaborators [55] reviewed the zooarchaeological literature and concluded that osteological records of *P.leo persica* have been recovered from the Northern coastal region of the Black Sea and Southern Ukrainian regions. According to the authors, the oldest Ukrainian remains correspond to the zooarchaeological sites of Bolhrad and Molukhov Bugor (6.4–5.0 kyr ago). Although some historical sources indicate *P. leo persica* persisted until the Middle Ages in Ukraine, to this date, no zooarchaeological evidence has been recovered [55]. Only three additional species of large-to-medium-size carnivores lived in this region: brown bears (*Ursus arctos*), grey wolves (*Canis lupus*), and, to a lesser extent, leopards (*P. pardus*; 55). Of the four aforementioned species of predators, it is possible that due to their hunting behavior, distribution range, and population size, only *P. leo persica* and *C. lupus* posed a potential threat to *E. ferus*.

In contrast, the number and diversity of extinct large carnivorans (see Table 3) that occupied a similar ecology as zebras is considerably higher relative to environments inhabited by ancient horses. Consequently, the predation pressure on *E. ferus* (the ancestor of the extant domesticated horse) was considerably lower compared to extant *E. grevyi*, *E. quagga*, and *E. zebra*. It is reasonable to consider that CM as a cardiac vulnerability evolved in these African equids as a byproduct of intense or acute response to predation or capture.

According to Olsen [56], Paleolithic sites associated with the foraging and hunting behaviors of *Homo sapiens* indicate that equids were frequently consumed in Europe. For example, 48% of 21 Upper Paleolithic Russian sites feature horse bones. Similarly, at the paleoanthropological site of Solutré in France, Paleolithic hunters (50–12 kyr ago) chased wild horses to a limestone cul-de-sac and killed them with spears and other hunting tactics [56]. Moreover, according to Olsen, specific horse hunting locations were used by multiple Paleolithic cultures through time (such as Aurignacian, Gravettian, Solutrean, and Magdalenian). As a result, European equine populations declined considerably by the Late Pleistocene and the Early Holocene. Olsen’s review of the literature indicated that by the Neolithic, small equine populations persisted in Western Europe (France), Central Europe (Austria, Germany, Czech Republic, Hungary, and Slovakia), Southern Europe (Moldova and Romania), and Eastern Europe (Ukraine). In particular, horse bones recovered from Western European sites comprised 0.2% of the average proportion of bone assemblages. Other sources indicate slightly higher percentages of horse remains in bone assemblages. Anthony [57] compiled data from multiple Holocene sites and computed the following estimates for the percentage of wild horses in Europe: (1) Iberian peninsula: less than 5%; (2) Western Europe: less than 5% to less than <10%; (3) Northern Europe: more than 10%; (4) Southern Europe: less than 5%; (5) Eastern Europe: more than 40%. As these percentages are based on remains recovered from human sites, additional studies are required to determine whether the variation in the percentage of wild horse bones across Europe results from differential hunting pressures or differences in horse populations. These hypotheses are not mutually exclusive in that specific Holocene sites featuring a high percentage of horse remains could be attributable to both frequent hunting and sizeable horse populations. Noticeable differences exist in the frequency of large mammalian predators in various Old World locations, suggesting that some regions operated as ecological safe havens for horse populations that did not evolve CM.

Table 5 features a non-exhaustive list of equine and large mammalian carnivorans disaggregated by regions during the Pleistocene, as well as a set of extant carnivoran predators of equine species. A bivariate correlation between the proportion of equine predator loss and the presence of CM was sizeable and reached statistical significance (*r* = −0.972, *p* = 0.0012), suggesting CM evolved in equine species inhabiting regions with persistent equine predators.

**Table 5:** A non-exhaustive list of equine and large carnivoran species across various Old World Pleistocene regions and a set of extant equine carnivoran predators.

Pleistocene Large Carnivorans and Equines Species							
Taxon	North Africa	East Africa	South Africa	Levant	Arabia and Iran	Europe	Number of Regions
*Lycaon pictus*	x	x	x				3
*Lycaon magnus*	x						1
*Canis lupus*				x	x	x	3
*Hyaena spelaea*						x	1
*Crocuta crocuta*	x	x	x	x	x		5
*Hyaena hyaena*	x	x		x	x		4
*Acinonyx jubatus*	x		x				2
*Homotherium* sp.	x						1
*Megantereon* sp.			x				1
*Panthera spelaea*						x	1
*Panthera leo*	x	x	x	x	x	x	6
*Panthera pardus*	x	x	x	x	x		5
*Ursus arctos*	x			x	x	x	4
*Ursus bibersoni*	x						1
*Canis simiensis*	x						1
*Equus africanus*	x	x		x			3
*Equus algericus*	x						1
*Equus capensis*			x				1
*Equus grevyi*		x					1
*Equus lylei*			x				1
*Equus mauritanicus*	x						1
*Equus melkiensis*	x						1
*Equus quagga*		x	x				2
*Equus zebra*			x				1
*Equus ferus*				x	x	x	3
*Equus hemionus*				x	x		2
*Equus hydruntinus*				x			1
**Extant Equine** **Predators**	***Equus zebra***	***Equus grevy***	***Equus quagga***	***Equus*** ***africanus***	***Equus hemionus***	***Equus*** ***kiang***	***Equus ferus***
*Canis simensis*				x			
*Panthera leo*	x	x	x	x			
*Panthera pardus*	x	x	x				
*Acinonyx jubatus*	x	x	x				
*Crocuta crocuta*	x		x				
*Lycaon pictus*	x	x	x				
*Hyena hyena*		x					
*Canis lupus*					x	x	x
**Region**	**North Africa**	**East Africa**	**South Africa**	**Levant**	**Arabia and Iran**	**Europe**	**Mean**
Number of Pleistocene Carnivorans	11	5	6	5	6	5	6.33
Number of Extant Equine Predators	2	4	6	0	1	1	2.33
Percentage of Equine Predator Loss	81.82	20.00	0.00	100.00	83.33	80.00	60.86
Number of Equine Species With CM	0	2	2	0	0	0	0.67

**Note**. Number of regions refers to the sum of biogeographical areas occupied by each species. Information collected from [51,53,55,58,59,60,61,62,63,64,65,66,67,68].

A recent study provides additional insight into the role of landscape visibility and predation risk among wild African equids. Kamaru and colleagues [69] examined the impact of bigheaded ant (*Pheidole megacephala*) invasions interfering with long-lasting mutualistic interactions between local trees and protective ant taxa (*Crematogaster* sp.). This ecological change increased the vulnerability of certain tree species to elephant foraging (*Loxodonta africana*) and led to severe deforestation. The authors discovered that areas impacted by elephant browsing, as a consequence of bigheaded ants displacing the protective ants, led to lower zebra mortality due to lion predation. The authors concluded that the reduction of trees caused a reduction of suitable ambush spots, decreasing the hunting success of lions inhabiting these altered environments. Consequently, zebra population density was higher in high visibility areas compared to uninvaded locations, while lion population density did not vary. Lions maintained this demographic stability by shifting their zebra-based diet to buffalo (*Syncerus caffer*). Extrapolating these findings, in conjunction with the paleoecological review provided above, indicates that the hunting success of large ambush predators varies as a function of landscape attributes such as vegetation density. Hence, open habitats, such as the past and present Eurasian Pontic-Caspian steppe, were suboptimal for sizable ambush predators, potentially reducing selective pressures acting on costly anti-predator behaviors (and CM) in the ancestors of extant horses.

Histological and pathological examinations offer additional insights into the connection between low predation and CM in wild (or feral) equids in Eurasian ecologies. Walzer and collaborators [70] provided a detailed review of mortality causes in Mongolian Przewalski’s (*E.c. prezwalski*) horses (1992 to 1999). The authors concluded that lung diseases comprised 29% of all deaths, followed by trauma and sandstorms (12%). The authors reported no exhaustion or cardiac disorders attributable to herding or handling. Robert and colleagues [71] provided additional information on pathologic patterns observed in Mongolian Przewalski’s horses. Per the differential diagnoses conducted in dead individuals (1998 to 2003), only two cases were classified as exhaustion, with only one of these instances corresponding to stressful conditions co-occurring with wasting and trauma. More recently, Kerekes and colleagues [72] examined the percentages of different causes of death in a sample of Przewalski’s horses inhabiting Hortobagy National Park in Hungary (1997 to 2018). According to the authors, of the 272 deaths, only 0.81% of the cases suffered from a cardiac condition. These results provide further evidence concerning the potential lack of CM in a species known to experience low mortality rates due to predation. This information is further described in Table 6.

**Table 6:** Causes of death and their corresponding mortality percentages in Przewalski’s horses are described by Waltzer and colleagues [70] and Kerekes and collaborators [72].

Cause of Death	Percentage [70]	Percentage [72]
Lung diseases	29%	
Sandstorms	12%	
Trauma	12%	
Drowning	8%	
Perinatal	8%	
Other	8%	
Transport	5%	
Cold related	5%	
Parturition/Unsuccessful delivery	5%	3.25%
Predation	3%	
Accident		39.02%
Shot		24.39%
Weakness		16.26%
Metabolic disorder		3.25%
Developmental disorder		3.25%
Miscarriage		2.44%
Pneumonia		2.44%
Bacterial		1.63%
Old age		1.63%
Eye tumor		0.81%
Food poisoning		0.81%
Heart condition		0.81%

### 4.2. Limitations and Future Directions

To maintain the required statistical power with the available phylogenetic sample, the current study was limited in that it could not examine a wide variety of the previously proposed domesticability traits, such as a species’ parent-offspring interactions, mating system, or habitat generalism. Future studies should find methods to examine a wider array of potential influences on domesticability, as well as the predictors in other animal clades such as carnivorans, rodents, birds, fish, and invertebrates. As comparative evidence on CM is predominantly described in ungulates and less in other mammalian and avian lineages, additional studies are needed to determine the extent to which CM occurs in other clades and its potential role in hindering domestication. For example, subsequent macroevolutionary comparative examinations could test the aforementioned implied *relaxed selection hypothesis*, wherein equids exposed to lower predation had an attenuated physiological response and thus were less susceptible to experience CM; therefore, they became more amenable to domestication. In a similar vein, additional evidence is required to determine the physiological pathway associated with relaxed selection for CM.

Another potential limitation pertains to our development of the DPI. As this measure is based on Zeder’s [3] and Larson and Burger’s [15] description of the domestication pathways, the values assigned to each species varied depending on the intentional action of human activities relative to indirect or accidental events leading to the domestication of wild taxa. A potential limitation of such an approach concerns attributing intentional actions based on archaeological, osteological, or biodemographic sources. Consequently, the validity of the DPI rests on current zooarchaeological reconstructions of human-animal symbiotic associations as described by Zeder [3] and Larson and Fuller [14]. This coding might change depending on new zooarchaeological findings challenging current descriptions of human-animal interactions, such as the extent to which humans intentionally domesticated specific taxa (i.e., the *directed pathway*). Moreover, some species have been classified under multiple pathways (e.g., pigs, *commensal*, and *prey pathways*) so that averages could be estimated across these multiple classifications. Subsequent studies should also consider the number of zooarchaeological publications per taxa, as some species have been studied more than others. The number of publications can be used as an indicator or research effort, a variable that should be controlled for in future statistical examinations. Another limitation of the DPI is that it does not disentangle historical processes leading to the various domestication pathways from the species’ actual phenotypes (e.g., domesticable vs. undomesticable traits). For example, the *directed pathway* depended on thousands of years of cultural innovations (including social complexity), as well as the animals’ phenotypic traits, facilitating the domestication process. Hence, despite the influence of historical processes on the *directed pathway*, species’ attributes (e.g., susceptibility to CM) can still promote or hinder domestication. Our analyses also counter Zeder’s view concerning the irrelevance of suitable traits for animals that were domesticated following the *directed pathway*. The macroevolutionary comparative evidence gathered in this study indicates that traits such as CM hindered the domestication process in ungulates, including those following the *directed pathway*, as indicated by the negative association between this cardiac condition and lower DPI scores. In other words, lower DPI scores reflecting the presence of more obstacles before reaching domestication are associated with the prevalence of CM (a phenotypic trait), even in species that were domesticated via the *directed pathway*.

This study operationalized domestication pathways as a series of ecological challenges to human societies that, in coevolution with animals amenable to domestication, they must overcome to achieve full domestication. The present examination, however, did not consider the internal social dynamics of human groups that may contribute significantly to the domestication process. Previous publications have recognized the role of community, social, and cultural ecologies in guiding domestication. For example, Hertler and colleagues [73] reviewed the ethnographic and archaeological literature on human-domesticate symbiosis and proposed several models concerning the mutualistic associations between humans and domesticated flora and fauna. The authors identified differences within networks of symbiotic associations among human groups, domesticated flora, and domesticated fauna across North America, Mesoamerican, South American, Eurasian, and Polynesian. More recently, Steklis and colleagues [74] examined the paleoanthropological and zooarchaeological literature on pre-Neolithic human sociopolitical complexity and its contribution to animal domestication. The authors argue that complex human societies featuring sophisticated ultrasocial institutions (social and political structures aimed at incentivizing cooperation and proscribing social defection among individuals who are distantly related at a genetic level; 74) emerged before the Neolithic Revolution and that the symbiotic associations established during domestication are an exaptation of human ultrasociality (i.e., extended ultrasociality). As societies differ in the diversity of their ultrasocial institutions, as well as their cultural variants and innovations, these differences are expected to contribute significantly to the domestication of plants and animals. Consequently, future empirical studies are required to consider the contribution of cultural and sociopolitical evolution to animal domestication across human biocultural groups.

## 5. Conclusions

Throughout the last two centuries, numerous scientific publications have described an array of phenotypic traits thought to facilitate animal domestication. The present study provided a phylogenetic comparative examination of a subset of predictor traits on a recently developed DPI (i.e., harder or easier pathway to domestication). Neither body mass, dietary breadth, nor gregariousness significantly predicted the DPI. Alternatively, CM had a negative and significant influence on DPI, suggesting CM hindered domestication. These results strongly suggest that, at least in ungulates, some of the phenotypic attributes often described in the literature as significant predictors of domestication did not have any significant contribution in the present phylogenetic comparative examinations, with the exception of the ‘Reaction to human’ trait (CM).

The Ancestral Character Reconstruction also suggested that some ungulate lineages have ancestral inclinations toward domestication while others have ancestral inclinations hindering domestication. To address the question “Why Were Zebras Not Domesticated?” these analyses also offer additional evidence concerning the macroevolutionary history of equine domestication. For example, in the case of the clade *Hippomorpha*, there appears to be an inclination toward domestication that preceded the Late Pleistocene-Early Holocene extinction. Indeed, our reconstructions counter the notion that low domesticability remained relatively unaltered across equine species, except for horses and donkeys. Instead, the analyses revealed that the last common ancestor of extant equine species featured moderate domesticability scores, facilitating the eventual domestication of horses and donkeys. This modest proclivity toward domestication in *Hippomorpha* eventually disappeared in zebras, a process likely attributable to the persistent predation pressures experienced by zebras in the Afrotropics compared to other equine species inhabiting more temperate regions that had fewer large land predators between the Late Pleistocene to the Early Holocene.

## Figures and Tables

**Table 2:** The phylogenetic generalized least squares model examines the influence of log body mass, diet breadth, and gregariousness and capture myopathy on DPI.

Predictor	Estimate	Std. Error	*t*-Value	*p*-Value
Intercept	0.778	0.491	1.59	0.1188
Log Body Mass	0.060	0.132	0.45	0.6542
Diet Breadth	−0.224	0.118	−1.89	0.0641
Gregariousness	0.304	0.166	1.83	0.0732
Capture Myopathy	−0.278	0.134	−2.08	0.0420

**Table 4:** Social systems of equids compared.

Social System		Domesticated Species	Undomesticated Species
**Social** **Organization**	Harem	Horses, sometimes Donkeys	Plains Zebra, Mountain Zebra, Kulan, Khulan, Gobi Kulan, Przewalski’s horse
	Fission-Fusion	Donkeys, sometimes Horses	Grevy’s Zebra, African wild asses (Nubian and Somali), Onager, Khur, Kiang (Western, Southern, and Eastern)
**Reproductive** **System**	Resource Defense Polygyny/ Multi-Male	Donkeys	Grevy’s Zebra, African wild asses (Nubian and Somali), Onager, Khur, Kiang (Western, Southern, and Eastern)
	Polygyny/ Single-Male	Horses	Plains Zebra, Mountain Zebra, Kulan, Khulan, Gobi Kulan, Przewalski’s horse
**Social** **Structure**	Dominance Hierarchy	Horses	Plains Zebra, Mountain Zebra, Przewalski’s horse, Kulan, Khulan, Gobi Kulan
	No Dominance Hierarchy	Donkey	Grevy’s Zebra, African wild asses (Nubian and Somali), Onager, Khur

## Data Availability

The original contributions presented in the study are included in the article, further inquiries can be directed to the corresponding author.
